# Predictors of expiratory flow limitation measured by forced oscillation technique in COPD

**DOI:** 10.1186/1471-2466-14-23

**Published:** 2014-02-19

**Authors:** Masashi Mikamo, Toshihiro Shirai, Kazutaka Mori, Yuichiro Shishido, Takefumi Akita, Satoru Morita, Kazuhiro Asada, Masato Fujii, Takafumi Suda

**Affiliations:** 1 Department of Respiratory Medicine, Shizuoka General Hospital, 4-27-1 Kita-Ando, 420-0881 Aoi, Shizuoka, Japan; 2 Second Department of Internal Medicine, Hamamatsu University School of Medicine, Hamamatsu, Japan

**Keywords:** Broadband, COPD, Expiratory flow limitation, Forced oscillation technique, Tidal breathing

## Abstract

**Background:**

Expiratory flow limitation (EFL) during tidal breathing is common in patients with severe COPD, and a major determinant of dynamic hyperinflation and exercise limitation. EFL can be measured by the forced oscillation technique (FOT); however, the relevance to clinical parameters is not fully understood. We hypothesized that emphysema extent and pulmonary function would contribute independently to the degree of EFL.

**Methods:**

Broadband frequency FOT and pulmonary function tests were performed in 74 patients with COPD to derive respiratory system resistance (Rrs) and reactance (Xrs), and the EFL index as expressed by the differences between inspiratory and expiratory phases of Xrs at 5 Hz (ΔX5). Emphysema extent was measured by high-resolution computed tomography and scored.

**Results:**

On the basis of the median value of ΔX5 (0.55 cmH_2_O/L/s), patients were classified into a high or low EFL index group. In multivariate regression analyses, a high EFL index was independently predicted by emphysema score, peripheral airway obstruction (forced expiratory flow between 25% and 75% of forced vital capacity), hyperinflation (functional residual capacity), and airway caliber (whole-breath Rrs at 5 Hz).

**Conclusions:**

EFL measured by FOT is a global measure of COPD that has separable etiologies and is useful for evaluating the disease condition.

## Background

Tidal expiratory flow limitation (EFL) occurs when an increase in transpulmonary pressure causes no increase in resting expiratory flow. This phenomenon is common in patients with severe COPD and is a major determinant of dynamic hyperinflation and exercise limitation [1,2]. Dellacà et al. indicated that the differences between inspiratory and expiratory phases of respiratory system reactance (ΔXrs) measured by the forced oscillation technique (FOT) allowed the detection of EFL [1]. It is supposed that Xrs normally reflects the elastic and inertial properties of the respiratory system but, with flow limitation, oscillatory signals cannot pass through the choke points and reach the alveoli. During EFL, respiratory system resistance (Rrs) and Xrs will reflect the mechanical properties of airways proximal to the choke points, which are much stiffer than the periphery, producing a marked reduction in apparent compliance and a fall in Xrs.

In contrast to the monofrequency FOT used by Dellacà et al., clinical application of the broadband frequency FOT has progressed recently with the spread of commercially available devices: the impulse oscillation system (IOS) [3] and MostGraph [4]. Several investigators have reported that ΔXrs, the EFL index, could discriminate between COPD and asthma [5-7], and between COPD and pulmonary fibrosis [8]; however, the relevance of EFL to clinical parameters is not fully understood. We hypothesized that emphysema extent and pulmonary function would contribute independently to the degree of EFL in patients with COPD. In this cross-sectional study we measured the EFL index by broadband frequency FOT in 74 patients with COPD and assessed the differences in clinical features between patients with a high and low EFL index.

## Methods

### Subjects

Seventy-four patients with COPD who attended outpatient clinics at Shizuoka General Hospital for routine check-ups between October 2009 and December 2012 were enrolled in this study. The patients satisfied the definition of the Global Initiative for Chronic Obstructive Lung Disease (GOLD) [9] and had been receiving medications, including long-acting antimuscarinic agents, long-acting β2-agonists, inhaled corticosteroids, or sustained-release theophylline. They were clinically stable and had had no exacerbations, defined as increased dyspnea associated with a change in the quality and quantity of sputum, for at least one month before the study.

Thirty-nine healthy control subjects without pulmonary diseases were recruited from our hospital staff. The protocols were approved by the Institutional Review Board of Shizuoka General Hospital (SGH 11-07-20) and informed consent was obtained from all subjects prior to the study.

### Modified Medical Research Council (mMRC) and COPD assessment test (CAT)

The mMRC scale was used to evaluate dyspnea in daily living, grading 0 (only get breathless with strenuous exercise) to 4 (too breathless to leave the house or breathless when dressing) [10].

The CAT (Japanese version, supplied by GlaxoSmithKline Japan) questionnaire consists of 8 items (cough, phlegm, chest tightness, breathlessness going up hills/stairs, activity limitations at home, confidence leaving home, sleep, and energy) assessing and quantifying the symptoms and impacts of COPD [11]. Each item is scored from 0 to 5 giving a total score range from 0 to 40, corresponding to the best and worst health status, respectively.

### Measurement of respiratory impedance and pulmonary function tests

On the same examination day, when their clinical symptoms were stable, measurements of respiratory impedance using FOT and pulmonary function tests were performed in that order. Short-acting β2 agonists were not used for more than 12 hours before these tests in every case.

Respiratory impedance was measured with broadband FOT using a commercially available device (MostGraph-01; Chest M.I. Co. Ltd., Tokyo, Japan) [4,7] and met standard recommendations [12]. Impulse oscillatory signals generated by a loud speaker at intervals of 0.25 seconds were applied to the respiratory system through the mouthpiece during tidal breathing at rest. Mouth pressure and flow signals were measured and calculated to obtain Rrs and Xrs properties against oscillatory frequency ranging from 4 to 36 Hz. During measurements, the subjects supported their cheeks firmly to reduce upper airway shunting while sitting with their neck in a comfortable neutral posture. Since the curves of Rrs and Xrs versus frequency could be obtained every 0.25 seconds, they were serially lined up against the time axis with assigned color gradients, resulting in colored 3-dimensional imaging patterns.

In the present study, we used Rrs at 5 and 20 Hz (R5 and R20, respectively), and the difference between R5 and R20 (R5-R20) as an indicator of the frequency dependence of Rrs, which is supposed to reflect inhomogeneous ventilatory mechanics [13]. We also used Xrs at 5 Hz (X5), which reflects elastic and inertial properties of the lung, resonant frequency (Fres) where Xrs crosses zero and the elastic and inertial forces are equal in magnitude and opposite, and a low-frequency reactance area (ALX), which is the integral of Xrs at 5 Hz to the Fres. Each oscillatory index is expressed as the mean values during a respiratory cycle (whole-breath), inspiratory and expiratory phases, and the differences between inspiratory and expiratory phases (Δ). In the present study, ΔX5 was used as the EFL index. Patients were classified into high or low EFL index groups according to the median value of EFL index.

Spirometry and lung volumes were determined using computerized equipment (model CHESTAC-8800; Chest M.I. Co. Ltd., Tokyo, Japan) according to the recommendations [14,15]. Predicted values for pulmonary function tests, excluding inspiratory capacity (IC), were obtained from the Japanese Respiratory Society guidelines [16] and those for IC were obtained from previous reports [17,18].

### Emphysema score

Emphysema was evaluated by high-resolution computed tomography (HRCT) according to the method reported previously [8,19]. Briefly, HRCT findings were evaluated at 3 anatomic levels in both lungs: near the superior margin of the aortic arch (level of the upper lung field), at the level of the carina (level of the middle lung field), and at the level of the orifice of the inferior pulmonary veins (level of the lower lung field). Emphysema was defined as a focal region of low attenuation without visible walls. Cysts were defined as round air spaces with a well-defined wall. Emphysema with cysts, if any, was scored visually in the 6 fields and summed. The score in each lung field was calculated according to the percentage of low-attenuation areas (%LAA): score 0, %LAA <5%; score 1, %LAA ≥5%– < 25%; score 2, %LAA ≥25%– < 50%; score 3, %LAA ≥50%– < 75%; and score 4, %LAA ≥75%. The threshold level between the normal lung density area and LAA was defined as -950 Hounsfield units on the basis of our previous study [8]. Thus, the total emphysema scores ranged from 0 to 24.

### Statistical analysis

Comparisons among groups were made using the Kruskal-Wallis test, followed by multiple comparisons among groups using the Mann–Whitney U test. The chi-square or Fisher’s exact test was used to test significance in group differences with respect to the percentage of patients in various categories. Correlations between variables were performed using the Spearman rank correlation coefficient. Multivariate logistic regression analyses were performed to adjust for effects among multiple variables for elevation of the EFL index (ΔX5). Model selection was made by the best subset selection procedure using Akaike’s information criteria (AIC). Stat View Version 5.0 (SAS Institute, Cary, NC, USA) and R version 2.15.2 (R Foundation for Statistical Computing, Vienna, Austria, 2012) were used for statistical calculations. A p value of <0.05 was considered significant, and all tests were 2 sided.

## Results

**Figure 1:**
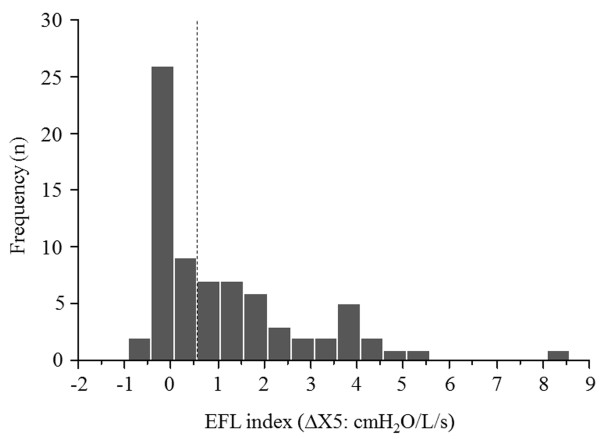
**Frequency distribution of the EFL index (∆X5) in 74 patients with COPD.** The patients were classified into high or low EFL index groups according to the median value of 0.55 (cmH_2_O/L/s) (dotted line). Abbreviations: ∆X5, difference between inspiratory and expiratory respiratory system reactance; EFL, expiratory flow limitation.

**Figure 2:**
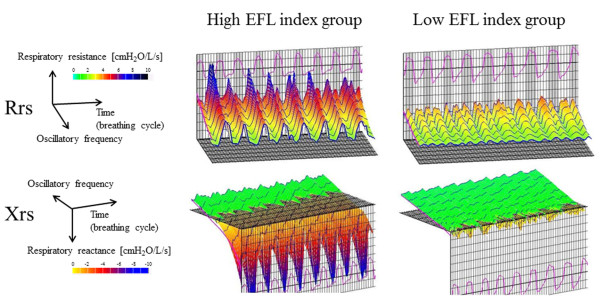
**Colored 3-dimensional images of Rrs and Xrs in each representative patient with high or low EFL index.** Respiratory cycle dependence (Rrs was higher and Xrs shifted more negative in the expiratory phases than in the inspiratory phases) and frequency dependence (Rrs increased at lower frequencies and fell with increasing frequencies) were marked in patients with high EFL index. In contrast, Rrs was moderate over the entire frequency and respiratory cycle, and Xrs shifted slightly negative in patients with low EFL index. Abbreviations: EFL, expiratory flow limitation; Rrs, respiratory system resistance; Xrs, respiratory system reactance.

The clinical characteristics of the subjects, pulmonary function tests, and forced oscillatory parameters are shown in Tables 1 and 2. The frequency distribution of the EFL index (∆X5) in 74 patients is shown in Figure 1. According to the median value of 0.55 (cmH_2_O/L/s), 74 patients with COPD were classified into two groups, with 37 patients each with a high and low EFL index. mMRC scale, functional residual capacity (FRC), residual volume (RV), total lung capacity (TLC), and RV/TLC were significantly higher and forced expiratory volume in 1 second (FEV1), forced vital capacity (FVC), FEV1/FVC, IC, and forced expiratory flow between 25% and 75% of FVC (FEF 25-75%) were lower in the high EFL index group than in the low EFL index group. Whole-breath, inspiratory, and expiratory R5, R20, R5-R20, Fres, and ALX, and ∆X5 were significantly higher and whole-breath, inspiratory, and expiratory X5, ∆(R5-R20), ∆Fres, and ∆ALX were lower in the high EFL index group than in the low EFL index group. There was no difference in CAT and emphysema scores between the 2 groups. Figure 2 shows typical colored 3-dimensional images of Rrs and Xrs in each representative patient with a high or low EFL index.

**Table 1:** Characteristics of the study subjects

	**Patients with COPD n = 74**	**High EFL index group n = 37**	**Low EFL index group n = 37**	**Controls n = 39**
Age (years)	73 (54 – 86)	73 (62 – 84)*	73 (54 – 86)*	35 (24 – 69)
Gender (male/female)	71/3	35/2*	36/1*	19/20
Body mass index (kg/m^2^)	21.1 (15.2 – 32.3)	21.6 (15.6 – 32.3)	20.8 (15.2 – 27.7)	22.0 (16.9 – 30.8)
Current/ex/never smoker	9/65/0	4/33/0*	5/32/0*	4/9/26
Pack years	53.1 (5.0 – 150.0)	62.0 (5.0 – 132.0)*	51.3 (7.5 – 150.0)*	0.0 (0.0 – 33.0)
mMRC scale	1 (0 – 4)	2 (0 – 4)†	1 (0 – 4)	NA
CAT score	12.5 (0 – 32)	14 (3 – 32)	11 (0 – 30)	NA
Emphysema score	10 (0 – 23)	12 (0 – 23)	10 (0 – 21)	NA
GOLD I/II/III/IV	9/31/20/14	1/9/15/12†	8/22/5/2	NA
LAMA	26	11	15	NA
LABA	4	2	2	NA
LAMA + LABA	26	15	11	NA
LAMA + ICS/LABA	13	7	6	NA
Sustained-release theophylline	24	18†	6	NA
FEV1 (% predicted)	52.6 (17.8 – 108.0)	36.8 (17.8 – 108.0)†*	67.5 (20.0 – 93.8)*	100.0 (76.9 – 118.5)
FVC (% predicted)	81.4 (34.9 – 144.0)	75.6 (34.9 – 144.0)†*	91.4 (42.5 – 114.9)*	102.7 (82.5 – 123.1)
FEV1/FVC (%)	50.1 (28.7 – 69.9)	42.0 (28.7 – 66.8)†*	59.2 (30.4 – 69.9)*	84.2 (73.5 – 97.7)
IC (% predicted)	83.0 (38.5 – 124.8)	78.4 (38.5 – 121.6)†*	86.7 (53.6 – 124.8)	94.2 (62.5 – 146.4)
FEF 25-75% (% predicted)	18.6 (5.7 – 57.4)	10.1 (5.7 – 37.7)†*	28.5 (6.3 – 57.4)*	83.4 (45.8 – 114.3)
FRC (% predicted)	100.1 (56.9 – 165.5)	111.9 (65.4 – 165.5)†	85.8 (56.9 – 123.4)*	107.9 (66.2 – 138.4)
RV (% predicted)	149.0 (34.4 – 332.0)	182.4 (94.0 – 332.0)†*	133.1 (34.4 – 213.6)*	94.4 (65.1 – 239.6)
TLC (% predicted)	109.0 (75.9 – 152.2)	116.9 (76.5 – 152.2)†	101.5 (75.9 – 133.2)	107.9 (84.2 – 147.5)
RV/TLC (% predicted)	118.2 (35.7 – 180.7)	135.5 (71.8 – 180.7)†*	106.2 (35.7 – 162.9)	112.3 (68.7 – 168.5)

Values are shown as a median (range) or numbers.

*Abbreviations*: *CAT* COPD assessment test, *EFL* expiratory flow limitation, *FEF 25-75%* forced expiratory flow between 25% and 75% of FVC, *FEV1* forced expiratory volume in 1 second, *FRC* functional residual capacity, *FVC* forced vital capacity, *GOLD* Global Initiative for Chronic Obstructive Lung Disease, *IC* inspiratory capacity, *ICS* inhaled corticosteroids, *LABA* long-acting β2-agonists, *LAMA* long-acting antimuscarinic agents, *mMRC* modified Medical Research Council, *NA* not applicable, *RV* residual volume, *TLC* total lung capacity.

*p <0.05 versus controls, †p <0.05 versus low EFL index group.

**Table 2:** Comparison of forced oscillatory parameters

	**Patients with COPD n = 74**	**High EFL index group n = 37**	**Low EFL index group n = 37**	**Controls n = 39**
R5 (cmH_2_O/L/s) Whole breath	4.36 (1.58 – 8.06)	5.09 (2.45 – 8.06)†*	3.51 (1.58 – 7.99)*	2.79 (1.15 – 5.70)
Inspiratory	3.80 (1.57 – 8.16)	4.80 (1.90 – 8.16)†*	3.11 (1.57 – 7.64)*	2.48 (1.01 – 5.03)
Expiratory	4.78 (1.60 – 10.45)	5.55 (2.86 – 10.45)†*	4.17 (1.60 – 8.34)*	3.04 (1.29 – 6.36)
ΔR5	-0.78 (-5.51 – 0.45)	-0.98 (-5.51 – 0.45)	-0.70 (-2.71 – 0.36)	-0.54 (-2.39 – 0.16)
R20 (cmH_2_O/L/s) Whole breath	3.21 (1.45 – 6.41)	3.59 (1.77 – 6.00)†*	2.86 (1.45 – 6.41)*	2.57 (1.20 – 4.20)
Inspiratory	3.03 (1.44 – 6.56)	3.38 (1.79 – 5.27)†*	2.56 (1.44 – 6.56)*	2.47 (1.14 – 3.76)
Expiratory	3.41 (1.52 – 7.66)	3.72 (1.73 – 7.66)†*	3.08 (1.52 – 6.26)*	2.69 (1.26 – 4.84)
ΔR20	-0.29 (-3.31 – 0.34)	-0.32 (-3.31 – 0.34)	-0.28 (-1.72 – 0.30)	-0.28 (-1.3 – 0.29)
R5-R20 (cmH_2_O/L/s) Whole breath	1.22 (-0.04 – 2.96)	1.51 (0.50 – 2.96)†*	0.64 (-0.04 – 1.83)*	0.25 (-0.76 – 1.76)
Inspiratory	0.82 (-0.10 – 2.89)	1.32 (0.11 – 2.89)†*	0.39 (-0.10 – 1.99)*	0.11 (-0.96 – 1.27)
Expiratory	1.42 (0.02 – 3.52)	1.99 (0.88 – 3.52)†*	0.78 (0.02 – 2.08)*	0.36 (-0.57 – 2.24)
Δ(R5-R20)	-0.50 (-2.20 – 0.32)	-0.68 (-2.20 – 0.30)†*	-0.31 (-1.05 – 0.32)	-0.29 (-1.15 – -0.04)
X5 (cmH_2_O/L/s) Whole breath	-1.23 (-6.92 – 0.17)	-2.77 (-6.92 – -0.48)†*	-0.42 (-3.50 – 0.17)*	-0.29 (-1.48 – 0.20)
Inspiratory	-0.78 (-3.85 – 0.09)	-1.57 (-3.85 – -0.11)†*	-0.42 (-3.24 – 0.09)*	-0.33 (-1.33 – 0.18)
Expiratory	-1.63 (-11.17 – 0.25)	-3.74 (-11.17 – -0.85)†*	-0.37 (-3.77 – 0.25)*	-0.23 (-1.64 – 0.21)
ΔX5	0.55 (-0.47 – 8.50)	1.80 (0.59 – 8.50)†*	-0.03 (-0.47 – 0.54)	-0.06 (-0.37 – 0.48)
Fres (Hz) Whole breath	13.74 (4.23 – 26.52)	19.61 (7.91 – 26.52)†*	7.97 (4.23 – 25.96)*	6.53 (4.03 – 14.89)
Inspiratory	10.87 (4.46 – 27.07)	16.10 (5.76 – 24.83)†*	8.16 (4.46 – 27.07)*	6.71 (4.05 – 12.36)
Expiratory	16.10 (4.00 – 28.20)	22.27 (10.06 – 28.20)†*	7.82 (4.00 – 24.85)*	6.30 (4.00 – 17.42)
ΔFres	-2.33 (-15.00 – 2.98)	-6.34 (-15.00 – -0.08)†*	0.14 (-5.74 – 2.98)	0.17 (-5.37 – 1.75)
ALX (cmH_2_O/L/s x Hz) Whole breath	7.44 (0.04 – 62.87)	22.16 (1.96 – 62.87)†*	1.42 (0.04 – 33.88)*	0.92 (0.01 – 7.64)
Inspiratory	3.54 (0.07 – 45.55)	10.60 (0.37 – 45.55)†*	1.48 (0.07 – 32.92)*	1.02 (0.01 – 6.47)
Expiratory	11.34 (0.00 – 106.35)	32.20 (3.54 – 106.35)†*	1.19 (0.00 – 34.83)*	0.74 (0.00 – 8.87)
ΔALX	-3.31 (-86.95 – 2.35)	-15.51 (-86.95 – 0.00)†*	0.05 (-5.73 – 2.35)	0.15 (-3.96 – 1.11)

Values are shown as a median (range).

*Abbreviations*: *ALX* integrated low-frequency reactance area, *Δ* difference between inspiratory and expiratory phases, *EFL* expiratory flow limitation, *Fres* resonant frequency, *R5 and R20* respiratory system resistance at 5 Hz and 20 Hz, *R5-R20* difference between R5 and R20, *X5* respiratory system reactance at 5 Hz.

*p <0.05 versus controls, †p <0.05 versus low EFL index group.

The control subjects were significantly younger, with no male dominance, more nonsmokers, fewer pack-years, and better pulmonary function; however, there was no difference in the body mass index. Concerning forced oscillatory parameters, the control subjects had lower whole-breath, inspiratory, and expiratory R5, R20, R5-R20, Fres, and ALX values and less negative X5 values than patients with COPD; however, there was no difference in ∆Rrs, ∆X5 (EFL index), ∆Fres, and ∆ALX values between COPD patients with a low EFL index and controls.

We constructed a model from variables as follows: age, smoking history (pack-years), symptoms (mMRC and CAT), emphysema extent, spirometry (FEV1, FVC, FEV1/FVC, IC, and FEF 25-75%), lung volumes (FRC, RV, TLC, and RV/TLC), and Rrs (whole-breath R5 and R20, and ∆R5 and ΔR20). The univariate correlations between ∆X5 (EFL index) and predictor variables are shown in Table 3. ∆X5 (EFL index) correlated positively with the mMRC scale, FRC, RV, TLC, RV/TLC, R5, and R20, and negatively with FEV1, FVC, FEV1/FVC, and FEF 25-75%, but not with age, pack-years, CAT and emphysema scores, IC, ∆R5, or ∆R20. Multivariate logistic regression analysis of the high EFL index (ΔX5) was performed using the selected model including 7 variables (AIC = 53.3, Table 4). The high EFL index was independently predicted by the emphysema score, FEF 25-75%, FRC, and whole-breath R5, but not by mMRC, FEV1/FVC, and ΔR5. Unlike univariate analysis, multivariate analysis revealed that severe emphysema related to the high EFL index.

**Table 3:** Univariate correlations between ∆X5 (EFL index) and predictor variables

	**Rho**	**p value**
Age (years)	0.087	0.4556
Pack-years	0.181	0.1217
mMRC scale	0.282	0.0161
CAT score	0.084	0.4712
Emphysema score	0.107	0.3609
FEV1 (% predicted)	-0.526	<0.0001
FVC (% predicted)	-0.365	0.0018
FEV1/FVC (%)	-0.518	<0.0001
IC (% predicted)	-0.212	0.0707
FEF 25-75% (% predicted)	-0.640	<0.0001
FRC (% predicted)	0.427	0.0003
RV (% predicted)	0.443	0.0002
TLC (% predicted)	0.245	0.0363
RV/TLC (% predicted)	0.448	0.0001
Whole-breath R5 (cmH_2_O/L/s)	0.669	<0.0001
∆R5 (cmH_2_O/L/s)	-0.211	0.0709
Whole-breath R20 (cmH_2_O/L/s)	0.528	<0.0001
∆R20 (cmH_2_O/L/s)	-0.007	0.9539

Values are the Spearman rank correlation coefficient.

*Abbreviations*: *CAT* COPD assessment test, *Δ* difference between inspiratory and expiratory phases, *R5 and R20* respiratory system resistance at 5 Hz and 20 Hz, *EFL* expiratory flow limitation, *FEF 25-75%* forced expiratory flow between 25% and 75% of FVC, *FEV1* forced expiratory volume in 1 second, *FRC* functional residual capacity, *FVC* forced vital capacity, *IC* inspiratory capacity, *mMRC* modified Medical Research Council, *NA* not applicable, *RV* residual volume, *TLC* total lung capacity.

**Table 4:** Multivariate logistic regression analyses for predicting high EFL index

**Variables**	**Adjusted odds ratio**	**95% confidence interval**	**p value**
mMRC scale	0.969	0.412 – 2.282	0.9432
Emphysema score	1.296	1.013 – 1.659	0.0395
FEV1/FVC (%)	1.197	0.980 – 1.461	0.0777
FEF25-75% (% predicted)	0.752	0.590 – 0.960	0.0220
FRC (% predicted)	1.108	1.039 – 1.181	0.0017
R5 (cmH_2_O/L/s)	3.426	1.470 – 7.983	0.0043
∆R5 (cmH_2_O/L/s)	0.423	0.134 – 1.342	0.1443

*Abbreviations*: *Δ* difference between inspiratory and expiratory phases, *R5* respiratory system resistance at 5 Hz, *EFL* expiratory flow limitation, *FEV1* forced expiratory volume in 1 second, *FVC* forced vital capacity, *FEF 25-75%* forced expiratory flow between 25% and 75% of FVC, *FRC* functional residual capacity, *mMRC* modified Medical Research Council.

## Discussion

We assessed whether emphysema extent and pulmonary functions contributed independently to the degree of EFL measured by broadband FOT in 74 patients with COPD. It was found that the high EFL index was independently predicted by emphysema extent as measured by HRCT, peripheral airway obstruction as expressed by FEF 25-75%, hyperinflation as expressed by FRC, and airway caliber as expressed by whole-breath R5. These results suggest that EFL measured by FOT is a global measure of COPD that has separable etiologies and therefore this could be used for stratifying patients and evaluating the severity of their disease or treatment response.

We consider respiratory impedance measured by FOT useful in research and clinical practice for the following reasons. First, it is performed during tidal breathing and does not require specific maneuvers or noticeable interference with respiration. Secondly, it provides information that is applicable to resting conditions or daily activities. Thirdly, it can be performed under different breathing conditions, such as deep expiration, and so provides information about the mechanical properties of the respiratory system that can be complementary to spirometry. Recent studies indicate that not only whole-breath but within-breath analyses of respiratory impedance bring useful information on the pathophysiology of COPD and asthma. On the basis of the report by Dellacà et al. that ΔX5 is a surrogate marker for EFL, we found that within-breath changes in Xrs (ΔX5 and ΔFres) discriminated between patients with COPD and asthma [7]. Other investigators obtained similar results with the IOS, a slight different method of FOT from the method in the present study [5,6]. Thus, these results indicate that EFL measured by FOT is a useful measure to diagnose COPD.

In the present study, we confirmed that the emphysema extent as measured by HRCT was an independent predictor of the degree of EFL (ΔX5), suggesting that reduced lung elastic recoil due to emphysema may be a cause of EFL. We previously showed that ΔX5 values in 86 patients with COPD were significantly higher than those in 45 patients with pulmonary fibrosis, possibly because the latter has increased lung elastic recoil (mean, 1.23 vs -0.18 cmH_2_O/L/s, respectively: p <0.05) [8]. There have been only a few published studies concerning the relationship between respiratory impedance and emphysema on HRCT. Crim et al. found a poor relationship (Pearson’s r ≤0.16) between emphysema extent and Rrs and Xrs measured by IOS in a large study but they did not analyze within-breath changes [20]. Timmins et al. also reported that there was no correlation between emphysema extent and ΔX6 measured by their in-house monofrequency FOT device [21]. Consistent with these reports, there was no correlation in univariate analysis in the present study, but multivariate analysis revealed that severe emphysema related to the high EFL index, indicating the need to control for confounding factors.

We also confirmed in the present study that whole-breath R5 was the strongest predictor of the degree of EFL (ΔX5), but not ΔR5. Rrs reflects dissipative mechanical property of the lung [12], in other words, viscous resistance. As airway obstruction increases in COPD, Rrs rises and becomes more frequency dependent, especially at lower frequencies [22], implying that R5 is supposed to be a measure of airway caliber. Timmins et al. also found a strong correlation between R6 and EFL index [21], while Dellacà et al. confirmed that ΔR5, in contrast to ΔX5, was not associated with EFL [1].

There was an independent association between FEF 25-75% and EFL index; the lower the FEF 25-75%, the higher the EFL index. When the flow-volume curve becomes more concave because of highly reduced flow in the effort-independent part of the curve, the degree of EFL increases; however, there was no association between FEV1/FVC and EFL index in multivariate analysis. These results suggest that the EFL index measured by FOT during tidal breathing reflects peripheral airway obstruction as measured by forced expiration. FRC, a measure of hyperinflation, was also an independent predictor of the EFL index in the present study, whereas Timmins et al. found no correlation. Possible explanations include the difference in the sample size or the FOT method.

We classified the patients into high or low EFL index groups according to the median value of the EFL index (0.55 cmH_2_O/L/s) of patients with COPD. Although all the control subjects had lower values than this cutoff level, this was not a genuine threshold value for detecting EFL. Dellacà et al. established a threshold value of 2.8 cmH_2_O/L/s for the detection of EFL, which was proven by esophageal manometry with high sensitivity and specificity [1]; however, our results were obtained with a broadband FOT device, MostGraph, whereas Dellacà et al. used their in-house monofrequency FOT. Therefore, the threshold value identified by Dellacà et al. may not be applicable to our patients and would have to be established for MostGraph by further studies.

Since the FOT device used in the present study can provide colored 3-dimensional images of respiratory impedance [7,23], the difference in the images between patients with a high and low EFL index was distinct. This technique enables rapid perception of the disease condition rather than comparing each value, and would be useful if applied more widely in real clinical practice.

GOLD recommends the use of the mMRC scale or CAT to assess symptoms in patients with COPD; however, there was a difference in the analysis between these parameters in the present study. The univariate correlations in Table 3 showed that ∆X5 (EFL index) correlated positively with the mMRC scale, but not with CAT score. Similarly, mMRC scale was higher in the high EFL index group than in the low EFL index group, but there was no difference in CAT score between the 2 groups. A possible explanation may be that the mMRC is a unidimensional measurement to quantify only dyspnea whereas the CAT score is a multidimensional method, which assess 8 items; not only dyspnea but also other symptoms and health status [24].

Methods for assessing EFL include not only FOT but also negative expiratory pressure (NEP), both of which are simple, noninvasive, and practical techniques [2]. A previous study found a good agreement between FOT and NEP despite some differences in detection of EFL [25]; however, the relationship between these techniques and NEP has not been fully understood. Further studies are needed to clarify this matter.

## Conclusions

The risk of EFL is independently predicted by emphysema extent, peripheral airway obstruction, hyperinflation, and airway caliber. EFL measured by FOT is a global measure of COPD and is useful for evaluating the disease condition.

## Abbreviations

AIC: Akaike’s information criteria; ALX: Low-frequency reactance; CAT: COPD assessment test; ∆: Differences between inspiratory and expiratory phases; EFL: Expiratory flow limitation; FEF 25-75%: Forced expiratory flow between 25% and 75% of FVC; FEV1: Forced expiratory volume in 1 second; FOT: Forced oscillation technique; FRC: Functional residual capacity; Fres: Resonant frequency; FVC: Forced vital capacity; GOLD: Global Initiative for Chronic Obstructive Lung Disease; HRCT: High-resolution computed tomography; IC: Inspiratory capacity; ICS: Inhaled corticosteroids; IOS: Impulse oscillation system; LAA: Low-attenuation area; LABA: Long-acting β2-agonists; LAMA: Long-acting antimuscarinic agents; mMRC: Modified Medical Research Council; Rrs: Respiratory system resistance; R5: Rrs at 5 Hz; RV: Residual volume; TLC: Total lung capacity; Xrs: Respiratory system reactance.

## Competing interests

The authors declare that they have no competing interests.

## Authors’ contributions

MM and TS contributed to the data collection and analysis, and writing of the manuscript. KM contributed to the data collection, analysis, and interpretation, and writing of the manuscript. YS, TA, SM, KA, and MF contributed to the data collection. TS contributed to the critical review of the manuscript and final draft. All authors read and approved the final manuscript.

## Pre-publication history

The pre-publication history for this paper can be accessed here:

http://www.biomedcentral.com/1471-2466/14/23/prepub
